# Identification of Novel Mutations in *CDC20*: Expanding the Mutational Spectrum for Female Infertility

**DOI:** 10.3389/fcell.2021.647130

**Published:** 2021-04-09

**Authors:** Lin Zhao, Yichun Guan, Qingxia Meng, Weijie Wang, Ling Wu, Biaobang Chen, Jijun Hu, Jiawei Zhu, Zhihua Zhang, Jian Mu, Yao Chen, Yiming Sun, Tianyu Wu, Wenjing Wang, Zhou Zhou, Jie Dong, Yang Zeng, Ruyi Liu, Qiaoli Li, Jing Du, Yanping Kuang, Qing Sang, Lei Wang

**Affiliations:** ^1^Institute of Pediatrics, Children’s Hospital of Fudan University and Institutes of Biomedical Sciences, State Key Laboratory of Genetic Engineering, Fudan University, Shanghai, China; ^2^Department of Reproductive Medicine, The Third Affiliated Hospital of Zhengzhou University, Zhengzhou, China; ^3^Center for Reproduction and Genetics, The Affiliated Suzhou Hospital of Nanjing Medical University, Suzhou, China; ^4^Reproductive Medicine Center, Shanghai Ninth Hospital, Shanghai Jiao Tong University, Shanghai, China; ^5^NHC Key Lab of Reproduction Regulation (Shanghai Institute of Planned Parenthood Research), Fudan University, Shanghai, China; ^6^Obstetrics and Gynecology Hospital of Fudan University, Shanghai, China

**Keywords:** female infertility, *CDC20*, novel mutation, oocyte maturation arrest, fertilization failure

## Abstract

Oocyte maturation and fertilization are fundamental processes for successful human reproduction, and abnormalities in these processes will cause infertility. Recently, we identified biallelic mutations in *CDC20* that are responsible for human oocyte maturation arrest, fertilization failure, and early embryonic development arrest. In this study, we screened for further *CDC20* mutations in a new cohort of patients with abnormalities in oocyte maturation, fertilization, and early embryonic development. Through whole-exome sequencing, we identified the four novel mutations c.887G > A (p. Arg296Gln), c.964C > T (p.Arg322^∗^), c.1155G > C (p.Trp385Cys), and c.330 + 1G > A (p. Glu111Ilefs^∗^36) and one previously reported mutation c.965G > A (p.Arg322Gln) in *CDC20* in four infertile individuals from three independent families. The patients had different phenotypes of oocyte maturation arrest and fertilization failure resulting from the different mutations. This study confirms our previous research and expands the spectrum of known mutations in *CDC20*, providing new evidence supporting the function of *CDC20* in the genetic etiology of female infertility characterized by oocyte maturation arrest and fertilization failure.

## Introduction

Successful human reproduction requires normal oocytes maturation, fertilization, and embryonic development, and thus any defects in the processes of oocyte maturation and fertilization will cause female infertility. Assisted reproduction technology (ART) is an effective treatment for infertility in which mature oocytes are fertilized with sperms through co-incubation called *in vitro* fertilization (IVF), or through direct injection of a single sperm under microscopic visualization, called intracytoplasmic sperm injection (ICSI; Palermo et al., 1992; Myers et al., 2008). With the help of ART, the morphology of the immature, unfertilized oocytes or arrested embryos can be evaluated, which gives an opportunity to study the genetic causes behind these abnormalities.

In recent years, we and others have identified mutations in *TUBB8* (HGNC:20773), *PATL2* (HGNC:33630), and *TRIP13* (HGNC:12307) as being responsible for human oocyte maturation arrest and have identified biallelic mutations in *TLE6* (HGNC:30788) and *WEE2* (HGNC:19684) that cause human fertilization failure (Alazami et al., 2015; Feng et al., 2016a; Chen et al., 2017b; Sang et al., 2018; Zhang et al., 2020). Recently, we demonstrated that biallelic mutations in *CDC20* (HGNC:1723) are responsible for abnormalities in human oocyte maturation, fertilization, and early embryonic development (Zhao et al., 2020b). Despite these findings, the genetic basis for a large number of infertile patients remains to be elucidated.

CDC20, a mitotic activator of anaphase-promoting complex/cyclosome (APC/C), acts as a major downstream target for inhibition by the spindle assembly checkpoint (Hwang et al., 1998), and inhibition of CDC20 prevents the separation of sister chromatids until the microtubules radiating from the spindle poles are correctly attached to the kinetochores during both mitotic and meiotic processes (Musacchio and Hardwick, 2002; Musacchio and Salmon, 2007; Ge et al., 2009). It has been reported that *Cdc20* hypomorphic female mice are infertile or subfertile due to the failure of the first few embryonic divisions (Jin et al., 2010). In 2017, the mutations of *CDC20* have been reported to be associated with idiopathic azoospermia (Li et al., 2017). Until 2020, the explicit causal relationship between *CDC20* mutations and female infertility was established by our group. In that study, we identified several mutations that impaired the normal function of *CDC20*, which resulted in female infertility due to abnormal oocyte maturation, fertilization, or early embryonic development (Zhao et al., 2020b).

In light of the important role of CDC20, we screened for novel *CDC20* mutations in a new cohort of infertile female patients with abnormalities in the processes of oocyte maturation, fertilization, and early embryonic development. We identified four novel mutations and one previously reported mutation in *CDC20* in four infertile individuals from three independent families. These findings confirm our previous study and expand the mutational spectrum of *CDC20* that are responsible for female infertility.

## Materials and Methods

### Clinical Samples

A new cohort of 465 infertile female patients with oocyte maturation arrest, fertilization failure, and early embryonic arrest were recruited from the Suzhou Municipal Hospital, the Reproductive Medicine Department of the Third Affiliated Hospital of Zhengzhou University, and the Ninth Hospital affiliated with Shanghai Jiao Tong University and were used in this study. The patient recruit criteria were applied as follows: (1) female patients are younger than 45 years old, failing to conceive after 1 year (or longer) of regular unprotected sex, (2) had undergone ≥2 failed attempts of IVF/ICSI, characterized by oocyte maturation arrest, fertilization failure, or early embryonic arrest in IVF/ICSI attempts, (3) female patients with other known causes of infertility, including male factors, chromosome anomalies, radiotherapy, or chemotherapy, were excluded. Peripheral blood samples were taken for DNA extraction. This study was approved by the Ethics Committee of the Medical College of Fudan University and the Reproductive Study Ethics Committees of the hospitals.

### Ovarian Stimulation, Hormone Measurement, and Embryo Culture

The details for ovarian stimulation, oocyte retrieval, IVF/ICSI procedures, and embryo culture have been described elsewhere (Huang et al., 2019). hMG (Anhui Fengyuan Pharmaceutical) was started on day 3 of the menstrual cycle. Urinary hCG (Lizhu Pharmaceutical Trading Co., China) was administered at a dose of 5,000 IU when two or more follicles measured 18 mm or more. Serum FSH, LH, E_2_, and P levels were analyzed on spontaneous menstrual cycle day 3 (MC_3_) and the day of hCG triggering. Oocytes retrieval was scheduled at 34–36 h after trigger and IVF or ICSI was performed 4–5 h after oocytes retrieval.

For IVF, collected oocytes were incubated in human tubal fluid (HTF; Irvine Scientific, United States), supplemented with 10% serum substitute supplement (SSS; Irvine Scientific, United States), and 300,000 progressively motile spermatozoa and left overnight; For ICSI, denudated oocytes were injected with a single mechanically immobilized sperm and directly thereafter cultured in fertilization medium (HTF + 10% SSS). All embryos were maintained in Continuous Single Culture of HTF (Irvine Scientific, United States) and were incubated under oil at 37°C and a 5% O_2_ and 6% CO_2_ humidified incubators with 30 μL of culture media drop throughout the entire duration of *in vitro* culture.

### Genetic Studies

Genomic DNA was extracted from peripheral blood using the QIAamp DNA Blood Mini Kit (Qiagen). Whole-exome capture was performed using the SeqCap EZ Exome Kit (Roche), and sequencing was performed on the Illumina NovaSeq 6000 platform (Illumina). Sequencing analysis was compared with the human reference sequence (NCBI Genome build GRCh37). Mutations were annotated with GRCh37 and the dbSNP (version 138) and genome Aggregation Database (gnomAD) databases along with our in-house exome database, and functional prediction was performed with the SIFT and MutationTaster programs. For patient from consanguineous family, homozygosity mapping was performed with Homozygosity Mapper (Brazas et al., 2009). All candidate mutations were screened with the following criteria: (1) homozygous mutations with a minor allele frequency of less than 0.1% in gnomAD database and located within homozygous regions greater than 2.0 Mb, (2) predicted to be damaging or disease causing by SIFT or MutationTaster, (3) no homozygous or compound-heterozygous mutations in our control sample, and (4) inherited from both the father and mother if parents’ blood samples were available. The candidate genes were confirmed by Sanger sequencing of the affected probands as well as their parents and siblings.

### TA Clone Construction

The sequence of the compound heterozygous mutation (c.1155G > C and c.330 + 1G > A) was amplified from the genomic DNA of the proband (II-1) in family 3 and cloned into the pClone007 vector according to the manufacturer’s protocol (Tsingke).

### Expression Vector Construction

The full-length coding sequence of wild-type human *CDC20* (GenBank: NM_001255.3) was amplified from human metaphase I (MI) oocyte cDNA and cloned into the pCMV6-empty vector. Point mutagenesis was performed with the KOD-Plus Mutagenesis Kit (Toyobo) according to the manufacturer’s protocol. The FLAG tag was inserted into the wild type and mutant vectors at the C-terminus for the localization experiment.

### Cell Culture and Transfection

Chinese hamster ovary (CHO) cells were cultured in Dulbecco’s modified Eagle medium supplemented with 10% fetal bovine serum and 1% penicillin/streptomycin (Gibco) and maintained in a humidified incubator at 37°C with 5% CO_2_. Wild type and mutant plasmids of *CDC20* were transfected into CHO cells using the PolyJet *In Vitro* DNA Transfection Reagent (SignaGen) according to the manufacturer’s instructions.

### Western Blotting

The CHO cells were seeded in a six-well plate 1 day prior to transfection and then transfected with 1 μg of the plasmids. Cells lysates were acquired in RIPA lysis buffer (Shanghai Wei AO Biological Technology) with 1% protease inhibitor cocktail (Bimake). The protein concentration was measured by the bicinchoninic acid method, and equal amounts of protein were resolved on 10% SDS-PAGE gels and transferred to nitrocellulose filter membranes. Membranes were blocked in 5% non-fat milk for 1 h and incubated overnight at 4°C with primary antibodies. Antibodies against CDC20 (14866, CST) and cyclin B1 (55004-1-AP, Proteintech) were used at 1:1,000 dilution, and anti-vinculin (1:3,000 dilution, 13901, CST) was used as the internal control. Goat anti-rabbit IgG (1:5,000 dilution, M21001, Abmart) was used as the secondary antibody to detect the primary antibodies. The blots were finally captured using ECL Western Blotting Substrate (Tanon) after incubation with the secondary antibodies (1:5,000 dilution, Abmart).

### Minigene Assay

The c.330 + 1G > A mutation was located at the donor splice-site of intron 2. The full sequence of exon 1, intron 1, exon 2, intron 2, exon 3, and intron 3 was amplified from the genomic DNA of the proband (II-I) in family 3, and the PCR product contained both the wild-type allele and the c.330 + 1G > A mutant allele due to the heterozygous status of this mutation. The PCR product was cloned into the minigene vector, and the wild type and c.330 + 1G > A mutant plasmids were transfected into HeLa cells. After incubation for 36 h, total RNA was extracted using the RNeasy Mini Kit (Qiagen), and cDNA was obtained with the PrimeScript RT reagent kit (Takara).

### Mouse Oocyte Collection and Microinjection

Germinal vesicle (GV) oocytes were collected from the ovaries of 7–8-week-old ICR mice. For long-term incubation, oocytes were cultured in Minimum Essential Medium (Gibco) with 10% fetal bovine serum and 1% penicillin/streptomycin (Gibco) in a humidified incubator at 37°C with 5% CO_2_. GV breakdown was prevented with 2.5 μM milrinone (Sigma). For the localization experiment, wild type and mutant FLAG-tagged *CDC20* cRNAs were microinjected into the cytoplasm of each mouse GV oocyte. The injected GV oocytes were released into milrinone-free medium and subsequently fixed for immunofluorescence at 7 h after release to collect the MI oocytes. For the small interference RNA (siRNA) experiment, *Cdc20* 5’UTR siRNA (si*Cdc20*, 5′-UGU UCG GGA GAG CUG AGU ATT-3′, 40 μM) was injected into GV oocytes and incubated for 24 h to knock down the endogenous Cdc20 level, which led to MI arrest. For the rescue experiment, wild-type *CDC20* cRNA (750 ng/μL) and mutant *CDC20* cRNA (750 ng/μL) were microinjected into the cytoplasm of each mouse GV oocyte at 6 h after siRNA injection. The first polar body (PB1) extrusion rate was recorded at 12 h after the oocytes were released into milrinone-free medium.

### Immunofluorescence

The mouse oocytes were fixed and stained as previously described (Wu et al., 2018). Anti-FLAG antibody (1:500 dilution, Sigma-Aldrich) was used for determining the CDC20 localization. The kinetochores were stained with an anti-Crest antibody (1:500 dilution, Immunovision), and Hoechst 33342 (1:700 dilution, BD) was used to label the DNA. Oocytes were mounted on glass slides, and images were captured on a confocal laser-scanning microscope (Leica).

## Results

### Clinical Characteristics of the Affected Individuals

**FIGURE 1:**
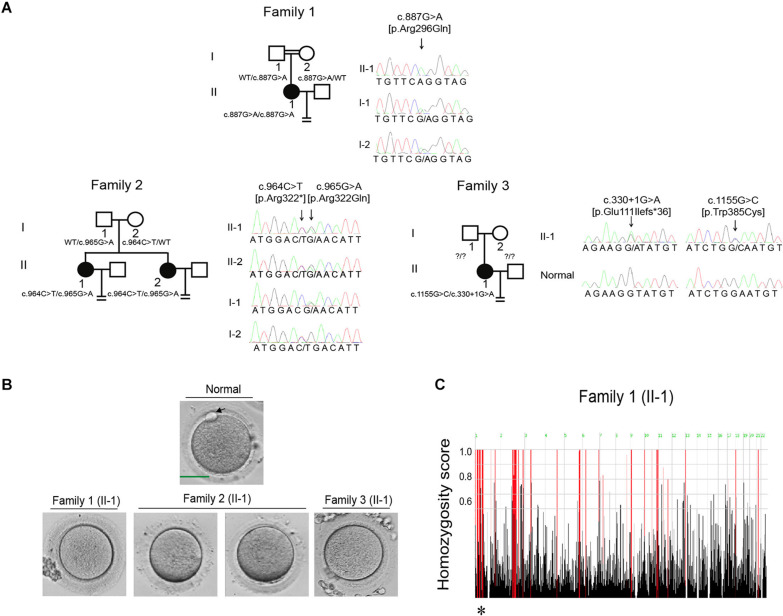
Pedigrees and the identification of mutations in *CDC20*. **(A)** Three pedigrees presented with oocyte maturation arrest and fertilization failure. Sanger sequencing confirmation is shown to the right of the pedigrees. Question marks indicate unavailable DNA samples. “Normal” represents the fertile control individual. Squares denote male family members, circles denote female members, black solid circles denote probands, the double lines between the parents in family 1 denote consanguineous marriage, and the equal sign denotes infertility. **(B)** The oocyte morphology of normal and affected individuals. The black arrow indicates PB1. Scale bar = 40 μm. **(C)** Homozygosity mapping of individual II-1 in family 1. Homozygous regions harboring the strongest signal are indicated in red, and the asterisk (^∗^) indicates the area where *CDC20* is located.

All three probands had been diagnosed with primary infertility of unknown cause for several years despite having normal menstrual cycles, and their male partners all had normal sperm counts and normal sperm morphology and motility. The detailed clinical information of ovarian stimulation characteristics is shown in Supplementary Table 1. The proband in family 1 was 33 years old and came from a consanguineous family (Figure 1A). She had undergone two failed IVF attempts during her 3 years of infertility, and all seven retrieved oocytes were arrested at the MI stage (Figure 1B). The proband in family 1 thus showed the phenotype of oocyte maturation arrest. The proband in family 2 was 37 years old and had been diagnosed with primary infertility for 11 years. She had undergone one failed IVF attempt and two failed ICSI attempts. In her IVF attempt, two oocytes were retrieved and both were arrested at the MI stage (Figure 1B). In her ICSI attempts, fourteen oocytes were retrieved, nine were arrested at the GV or MI stage, and five were maturated to the metaphase II (MII) stage. Only one MII oocyte was fertilized, but was arrested at the eight-cell stage. The proband in family 3 was 32 years old and had undergone one failed IVF attempt and one failed ICSI attempt. In her IVF attempt, five out of eight oocytes were MII oocytes. Only two of them were fertilized, but were they arrested at the one-cell stage. During her ICSI attempt, twelve out of thirteen oocytes were arrested at the MI stage, only one was matured failed to be fertilized. In brief, both the two probands in families 2 and 3 showed a mixed phenotype of maturation arrest and fertilization failure. The family pedigrees are shown in Figure 1A, and the clinical characteristics of the retrieved oocytes are summarized in Table 1.

**TABLE 1:** Oocyte and embryo characteristics of the IVF/ICSI attempts of the three probands.

Individual	Age (years)	Duration of infertility (years)	IVF/ICSI attempts	Total number of oocytes	GV oocytes	MI oocytes	PB1 oocytes	Fertilized oocytes	Embryos arrested at an early stage	Usable embryos
II-1 in family 1	33	3	IVF × 2	7	0	7	0	0	0	0
II-1 in family 2	37	11	IVF × 1	2	0	2	0	0	0	0
			ICSI × 2	14	2	7	5	1	1	0
II-1 in family 3	32	2	IVF × 1	8	1	2	5	2	2	0
			ICSI × 1	13	0	12	1	0	0	0

*IVF, in vitro fertilization; ICSI, intracytoplasmic sperm injection; GV, germinal vesicle; MI, metaphase I; PB1, the first polar body.*

### Identification of Novel Mutations in *CDC20*

**FIGURE 2:**
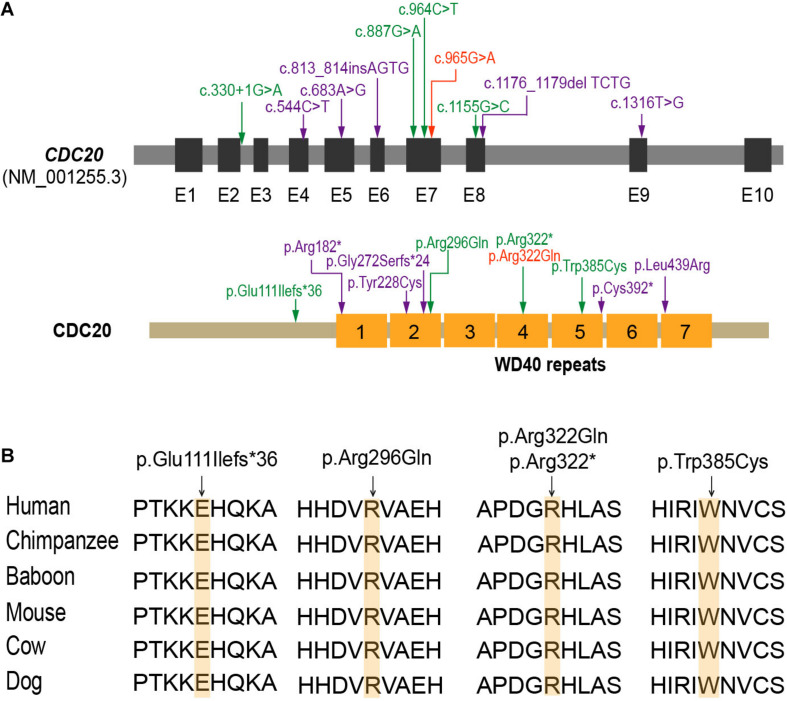
The location and conservation analysis of altered residues in *CDC20*. **(A)** The distribution of mutations in the *CDC20* gene and protein structure. The mutations described in our previous study (Zhao et al., 2020b) are marked in purple, the novel mutations identified in this study are marked in green, and mutation c.965G > A (p.Arg322Gln), which was found in both this study and our previous study, is marked in orange. **(B)** Conservation of the altered residues is indicated by the alignment of six mammalian species.

Mutational screening of *CDC20* (GenBank: NM_001255.3) was performed in our whole exome-sequenced cohort of patients with abnormalities in oocyte maturation, fertilization, and early embryonic development. We identified four affected individuals from three independent families with homozygous and compound heterozygous mutations in *CDC20* (Figure 1A). The proband (II-1) of family 1 came from a consanguineous family, and had the homozygous missense mutation c.887G > A (p. Arg296Gln) in *CDC20*. *CDC20* localized within the homozygous region (4.22M) of the proband in family 1 (Figure 1C), and the parents were both heterozygous carriers (Figure 1A). The functional impact of this mutation was assessed to be damaging by SIFT and disease causing by Mutation Taster (Table 2). The proband (II-1) of family 2 had a compound heterozygous *CDC20* mutation consisting of the missense mutation c.965G > A (p.Arg322Gln) and the nonsense mutation c.964C > T (p.Arg322^∗^). The parents were both heterozygous carriers, and the infertile sibling (II-2) carried the same compound heterozygous mutation as the proband (II-1) (Figure 1A). The proband (II-1) in family 3 carried a compound heterozygous mutation consisting of the missense mutation c.1155G > C (p.Trp385Cys) and the splicing mutation c.330 + 1G > A. Peripheral blood samples from the parents in family 3 were not available. To determine whether the compound mutations were inherited from both her father and mother separately, TA clone was constructed and Sanger sequencing was performed. As indicated in Supplementary Figure 1, the two mutations were located on different alleles, which confirms a recessive inheritance pattern. Information about the mutations is shown in Table 2, and the positions of the corresponding mutations and their conservation analysis in different species are shown in Figure 2.

**TABLE 2:** Overview of the *CDC20* mutations observed in the three families.

Family	Genomic position on Chr. 1 (bp)	cDNA change	Protein change	Mutation type	SIFT^*a*^	MutTas^*a*^	gnomAD^*b*^	gnomAD_eas^*b*^
1	43,826,442	c.887G > A	p. Arg296Gln	missense	D	D	3.2 × 10^–5^	0
2	43,826,519	c.964C > T	p. Arg322*	stop gain	NA	D	1.4 × 10^–5^	0
	43,826,520	c.965G > A	p. Arg322Gln	missense	T	D	8.5 × 10^–5^	9.0 × 10^–4^
3	43,825,310	c.330 + 1G > A	p. Glu111Ile fs*36	splicing	NA	NA	8.0 × 10^–6^	5.4 × 10^–5^
	43,826,868	c.1155G > C	p. Trp385Cys	missense	D	D	4.0 × 10^–6^	0

*^a^Mutation assessment by SIFT and MutationTaster (MutTas). T, tolerated; D, damaging or disease causing; NA, not available. ^b^Frequency of the corresponding mutations in the total and East Asian(eas) population of the gnomAD browser.*

### The Identified Novel Mutations Impaired the Normal Function of CDC20

**FIGURE 3:**
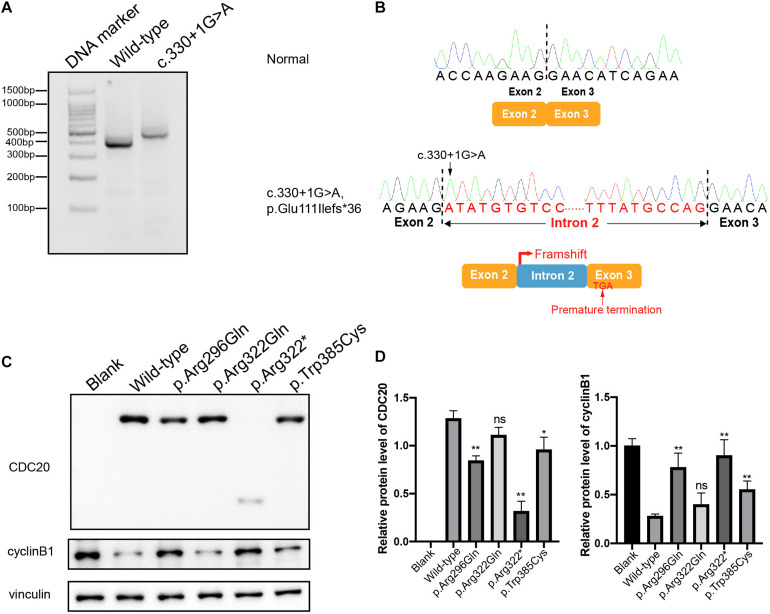
Effects of the mutations on *CDC20* transcript, CDC20 and cyclin B1 protein levels. **(A)** Agarose gel electrophoresis illustrating the effect of the *CDC20* c.330 + 1G > A mutation. Compared with the wild-type lane, the c.330 + 1G > A lane showed a larger size band. **(B)** The *CDC20* c.330 + 1G > A splicing mutation missed the canonical donor splice site, resulting in retention of intron 2 and the premature termination of the CDC20 protein. **(C)** The effects of the mutations on CDC20 and cyclin B1 protein level by western blotting in CHO cells transfected with wild type or mutant vectors. **(D)** Quantification of wild type and mutant CDC20 protein levels and cyclin B1 protein levels in CHO cells. Quantification was performed by measuring the band intensity of CDC20 and cyclin B1 relative to that of vinculin. The data are shown as means and SEM. *p < 0.05, **p < 0.01, ns, not significant.

The effect of the c.330 + 1G > A splicing mutation was studied with a minigene assay. Agarose gel electrophoresis showed a different-size band for the c.330 + 1G > A compared with the wild type, and the larger band indicated the abnormal alternative splicing isoform resulting from this mutation (Figure 3A). The cDNA sequence analysis of the alternative splicing isoform is shown in Figure 3B. The c.330 + 1G > A splicing mutation caused the canonical donor splice site missing, which resulted in the retention of intron 2 and the premature termination of CDC20 protein. This indicated that the splicing mutation c.330 + 1G > A affected the normal splicing of *CDC20* and resulted in an abnormal transcript.

To assess the impact of the missense mutations (p.Arg296Gln, p.Arg322Gln, and p.Trp385Cys) and the nonsense mutation (p.Arg322^∗^) on the function of CDC20, we first detected the CDC20 protein level in CHO cells. Compared with wild-type CDC20, the missense mutations p.Arg296Gln and p.Trp385Cys resulted in a reduction in the CDC20 protein level, while the nonsense mutation p.Arg322^∗^ resulted in a truncated protein (Figures 3C,D). Although the missense mutation p.Arg322Gln had no obvious effect on the CDC20 protein level in CHO cells, it was previously reported that the p.Arg322Gln mutation caused a reduction in the CDC20 protein level in the patient’s lymphoblastoid cell line *in vivo* (Zhao et al., 2020b).

During the metaphase to anaphase transition, Cdc20 binds to and activates the ubiquitin ligase activity of APC/C and enables the ubiquitination and degradation of cyclin B1, thus promoting the onset of anaphase (Nasmyth and Haering, 2005). We therefore further assessed the effect of the CDC20 mutations on cyclin B1 degradation. As shown in Figures 3C,D, overexpression of wild-type CDC20 significantly decreased the endogenous protein level of cyclin B1, while most of mutants affected the degradation of cyclin B1.

**FIGURE 4:**
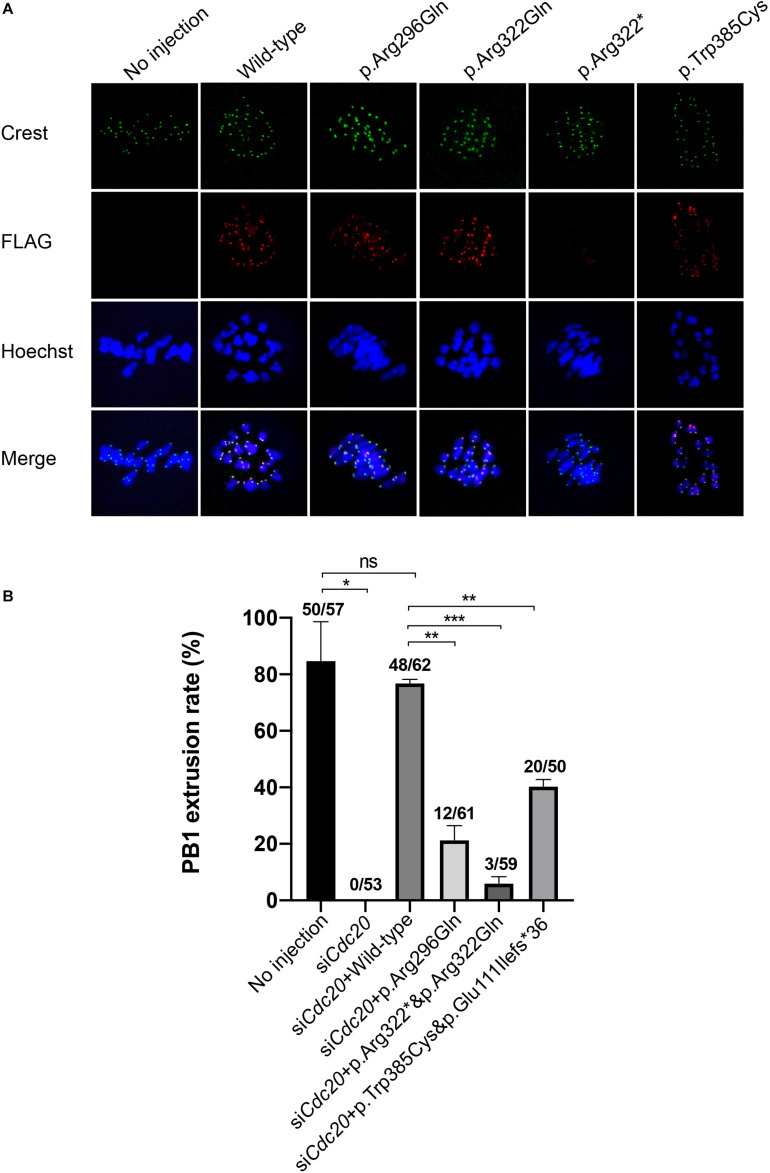
Effects of the mutations on kinetochore localization in mouse oocytes and the *CDC20* rescue ability in *Cdc20* knockdown mouse oocytes. **(A)** Kinetochore localization of wild type and mutant FLAG-tagged CDC20 in mouse MI oocytes. Images were captured by confocal microscopy (Leica). Hoechst and Crest were used to label the DNA and kinetochores, respectively. **(B)** The effects of the mutations on the rescue of *Cdc20* knockdown mouse oocytes. The number of oocytes with PB1 extrusion and the total number of oocytes used are listed at the top of the column. Significance was compared between the no injection group and the si*Cdc20* group, the si*Cdc20* coupled with wild-type *CDC20* cRNA group, and the si*Cdc20* coupled with mutant *CDC20* cRNA groups. Two independent experiments were performed followed the chi-square test. *p < 0.05, **p < 0.01, ***p < 0.001, ns, not significant.

It has been reported that Cdc20 localizes to the kinetochore in MI mouse oocytes (Tsurumi et al., 2004). To determine the effect of the mutations on human CDC20 localization, we expressed FLAG-tagged wild type and mutant CDC20 to monitor its subcellular localization in mouse oocytes. Wild-type CDC20 showed a strong kinetochore signal (Figure 4A), which was consistent with previous studies. For the two missense mutations (p.Arg296Gln and p.Trp385Cys), CDC20 also showed normal kinetochore localization similar to wild type. In contrast, CDC20 with the nonsense mutation (p.Arg322^∗^) failed to localize to the kinetochore (Figure 4A), indicating the functional impairment of CDC20 protein.

### Mutations in *CDC20* Impaired the Phenotypic Rescue in *Cdc20* Knockdown Mouse Oocytes

To further explore the effect of the mutations on CDC20 function in mouse oocytes, we performed a rescue experiment as described in our previous studies (Reis et al., 2007). The MI arrest phenotype in mouse oocyte was constructed by injecting *Cdc20* siRNA, and the PB1 extrusion could be rescued by supplementation with human wild-type *CDC20* cRNA (Figure 4B). Compared with wild-type, the homozygous or compound heterozygous mutations significantly reduced the ability of CDC20 to rescue the PB1 extrusion (Figure 4B). To assessed the further reason of reduced ability, we detected the CDC20 protein level of wild-type and mutations in mouse oocytes. Compared with wild-type CDC20, the missense mutation p.Arg296Gln, p.Trp385Cys and p.Arg322Gln resulted in a reduction on CDC20 protein level. The mutation corresponding to the splicing mutant (c.330 + 1G > A) p.Glu111Ilefs^∗^36 resulted in truncated protein, and the nonsense mutation p.Arg322^∗^ showed no detectable signal which indicates the severe impairment on protein level (Supplementary Figure 2). To further assess the effect of each individual mutant independently, the wild-type and mutant cRNA were injected separately into the MI arrest mouse oocyte which caused by injecting *Cdc20* siRNA. Compared with wild-type CDC20, all individual mutants significantly reduced the ability of CDC20 to rescue the PB1 extrusion, which indicates that each individual mutant affected the normal function of CDC20 (Supplementary Figure 3). All these results demonstrated that all the identified mutations affected the normal function of CDC20.

## Discussion

Here, we identified five mutations in *CDC20* in four infertile individuals with the phenotype of oocyte maturation arrest and fertilization failure. Among these, mutation c.965G>A (p.Arg322Gln) was reported in our previous study (Zhao et al., 2020b) while the other four mutations c.887G > A (p. Arg296Gln), c.964C > T (p.Arg322^∗^), c.1155G > C (p.Trp385Cys), and c.330 + 1G > A (p. Glu111Ilefs^∗^36) are novel mutations. Most of these mutations caused a decrease in CDC20 protein level and affected the degradation of cyclin B1 in CHO cells, and the splicing mutation c.330 + 1G > A (p. Glu111Ilefs^∗^36) caused an abnormal transcript. This study confirms the critical role of CDC20 in human reproduction.

We found phenotypic variability among the affected individuals with different *CDC20* mutations. The proband in family 1 had the phenotype of oocyte maturation arrest, while the probands in families 2 and 3 had a mixed phenotype of oocyte maturation arrest and fertilization failure. The different phenotypes of the patients in families 2 and 3 might be due to the different ovulation-inducing and fertilization treatments in their different IVF and ICSI attempts. Variable phenotypes were also found in our previous study of different *CDC20* mutations cause different phenotypes including oocyte maturation arrest, fertilization failure, and embryonic development problems (Zhao et al., 2020b).

Phenotypic variability caused by different mutations in the same gene has been reported in several studies (Feng et al., 2016b; Chen et al., 2017a, 2019; Zhao et al., 2020a, b), and the exact mechanism of this phenotypic variability is worthy of further study in a knock-in mouse model. In addition, cRNA injection has been shown to rescue the phenotype of infertile patients in our previous studies (Sang et al., 2018; Zhang et al., 2020; Zhao et al., 2020b). However, the safety of such interventions needs to be assessed comprehensively and might be further explored by using transgenic mice or primates.

Due to the coverage limitation of whole-exome sequencing (Bamshad et al., 2011), we could only identify single nucleotide changes or small fragment insertions or deletions on or near the exons, and long fragment-insertions and deletions, copy number variations, and mutations in the untranslated regions were undetectable. Further screening by whole-genome sequencing is worthy of further exploration and will help us to better understand the penetrance of the pathogenic genes more comprehensively.

In conclusion, we identified three novel biallelic mutations in *CDC20* that are responsible for female infertility, and this supports our previous study of *CDC20* mutants. Our study also expands the mutational spectrum of *CDC20* and provides genetic diagnostic marker for female infertility.

## Data Availability Statement

According to national legislation/guidelines, specifically the Administrative Regulations of the People’s Republic of China on Human Genetic Resources (http://www.gov.cn/zhengce/content/201906/10/content_5398829.htm, http://english.www. gov.cn/policies/latest_releases/2019/06/10/content_281476708945462.htm), no additional raw data is available at this time. Data of this project can be accessed after an approval application to the China National Genebank (CNGB, https://db.cngb.org/cnsa/). Please refer to https://db.cngb.org/, or email: CNGBdb@cngb.org for detailed application guidance. The accession code CNP0001687 should be included in the application. The data presented in the study are deposited in the China National Genebank (CNGB) repository, accession number (CNP0001687).

## Ethics Statement

The studies involving human participants were reviewed and approved by the Ethics Committee of the Suzhou Municipal Hospital, the Ninth Hospital affiliated with Shanghai Jiao Tong University, the Reproductive Medicine Department of the Third Affiliated Hospital of Zhengzhou University, and IRB of the Medical College of Fudan University. The patients/participants provided their written informed consent to participate in this study. The animal study was reviewed and approved by the Shanghai Medical College of Fudan University.

## Author Contributions

LWa and QS, and LZ conceived and designed the research study. YK, YG, QM, LWu, JH, and JZ contributed to the recruitment, characterization, and oocyte imaging of the patients. WeiW, YZ, and T W performed the microinjection in mouse oocytes. BC and JM contributed to the bioinformatics analysis. LZ performed the experiments. JDu provided the mouse keeping room. ZZho, YC, YS, WenW organized the medical records. ZZha, JDo, RL, and QL analyzed the data. LZ, LWa, and QS wrote the draft of this manuscript. All authors contributed to the article and approved the submitted version.

## Conflict of Interest

The authors declare that the research was conducted in the absence of any commercial or financial relationships that could be construed as a potential conflict of interest.

## Supplementary Material

Supplementary Figure 1 TA cloning and sequencing of patient II-1 in family 3. Sanger sequencing confirmation of a healthy individual and patient II-1 in family 3 indicates that the compound heterozygous mutation was located on two alleles, which confirms the recessive inheritance pattern. The two mutant bases are marked in red and framed in a red box.

Supplementary Figure 2 Effects of mutations on CDC20 protein level in the rescue experiment in mouse oocyte. **(A)** The effects of the mutations on CDC20 protein level by western blotting in mouse oocytes co-injected with *cdc20* siRNA and wild-type or mutant cRNA. **(B)** Quantification of wild-type and mutant CDC20 corresponding of Supplementary Figure 1A. Quantification was performed by measuring the band intensity of CDC20 relative to that of vinculin. The experiment was performed with two independent biological replicates yielding similar results. The data are shown as means and SEM. **p* < 0.05, ***p* < 0.01.

Supplementary Figure 3 Effects of the individual mutants on *CDC20* rescue ability in *Cdc20* knockdown mouse oocytes. The number of oocytes with PB1 extrusion and the total number of oocytes used are listed at the top of the column. Significance was compared between the no injection group and the si*Cdc20* group, the si*Cdc20* coupled with wild-type *CDC20* cRNA group, and the si*Cdc20* coupled with mutant *CDC20* cRNA groups. Experiments were performed followed the chi-square test. **p* < 0.05, ****p* < 0.001, *****p* < 0.0001, ns, not significant.
