# Supplementary figures and images for: Identification of Novel Mutations in CDC20: Expanding the Mutational Spectrum for Female Infertility
Source: Front Cell Dev Biol. 2021 Apr 9;9:647130. doi: 10.3389/fcell.2021.647130 (PMC8063106; doi:10.3389/fcell.2021.647130)

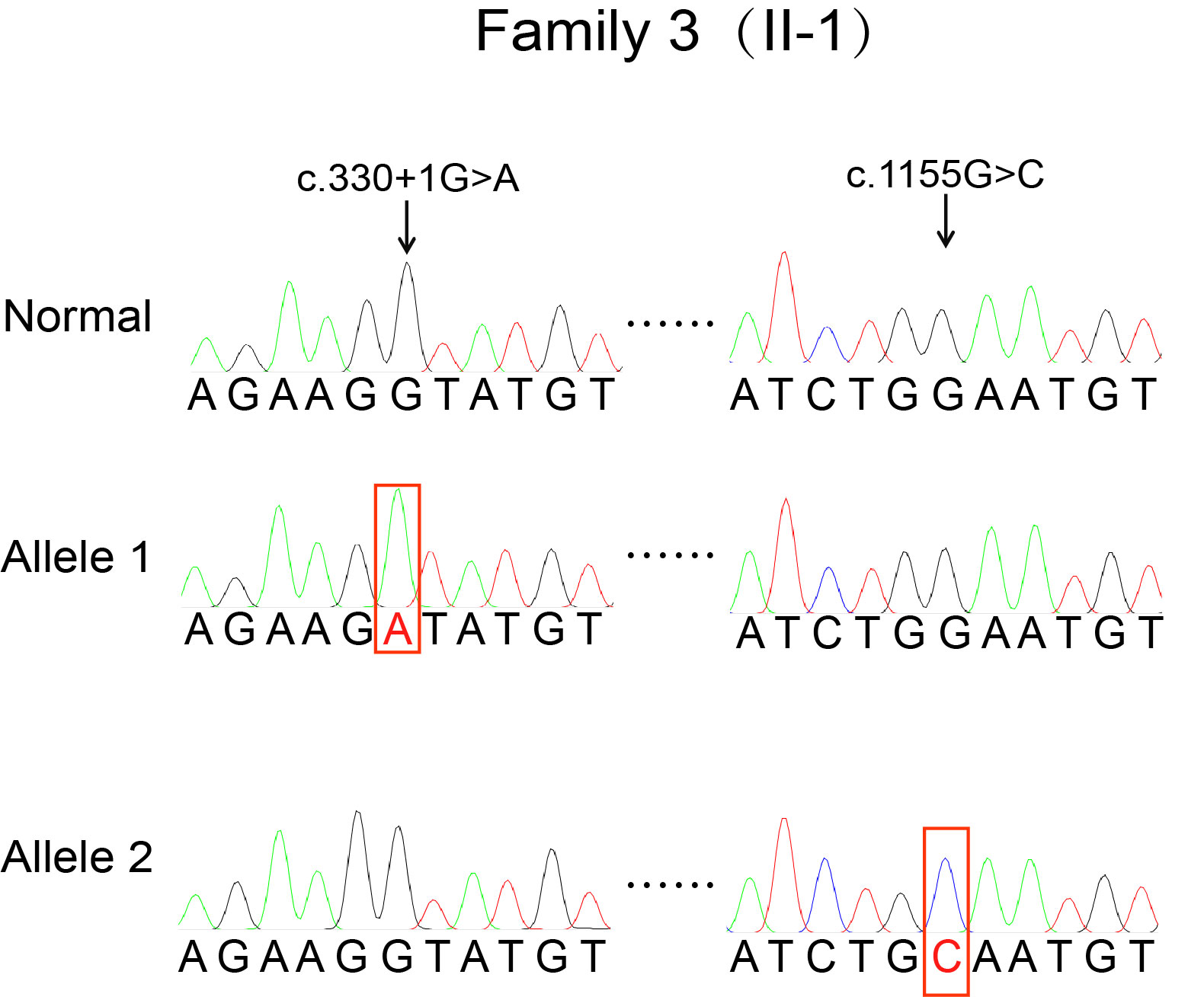

Supplement: Supplementary Figure 1 — TA cloning and sequencing of patient II-1 in family 3. Sanger sequencing confirmation of a healthy individual and patient II-1 in family 3 indicates that the compound heterozygous mutation was located on two alleles, which confirms the recessive inheritance pattern. The two mutant bases are marked in red and framed in a red box. [file Image_1.JPEG]

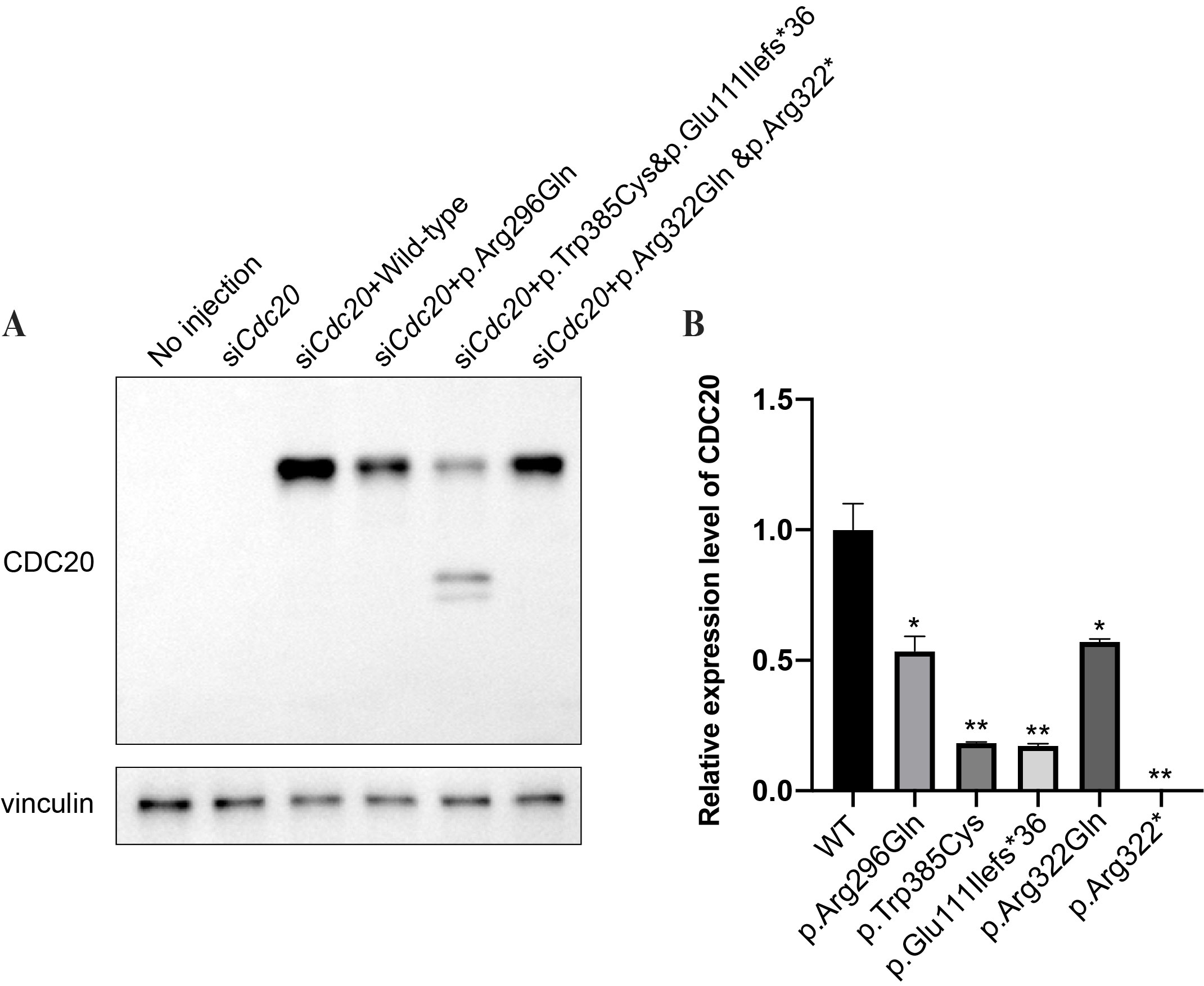

Supplement: Supplementary Figure 2 — Effects of mutations on CDC20 protein level in the rescue experiment in mouse oocyte. (A) The effects of the mutations on CDC20 protein level by western blotting in mouse oocytes co-injected with cdc20 siRNA and wild-type or mutant cRNA. (B) Quantification of wild-type and mutant CDC20 corresponding of Supplementary Figure 1A. Quantification was performed by measuring the band intensity of CDC20 relative to that of vinculin. The experiment was performed with two independent biological replicates yielding similar results. The data are shown as means and SEM. *p < 0.05, **p < 0.01. [file Image_2.jpg]

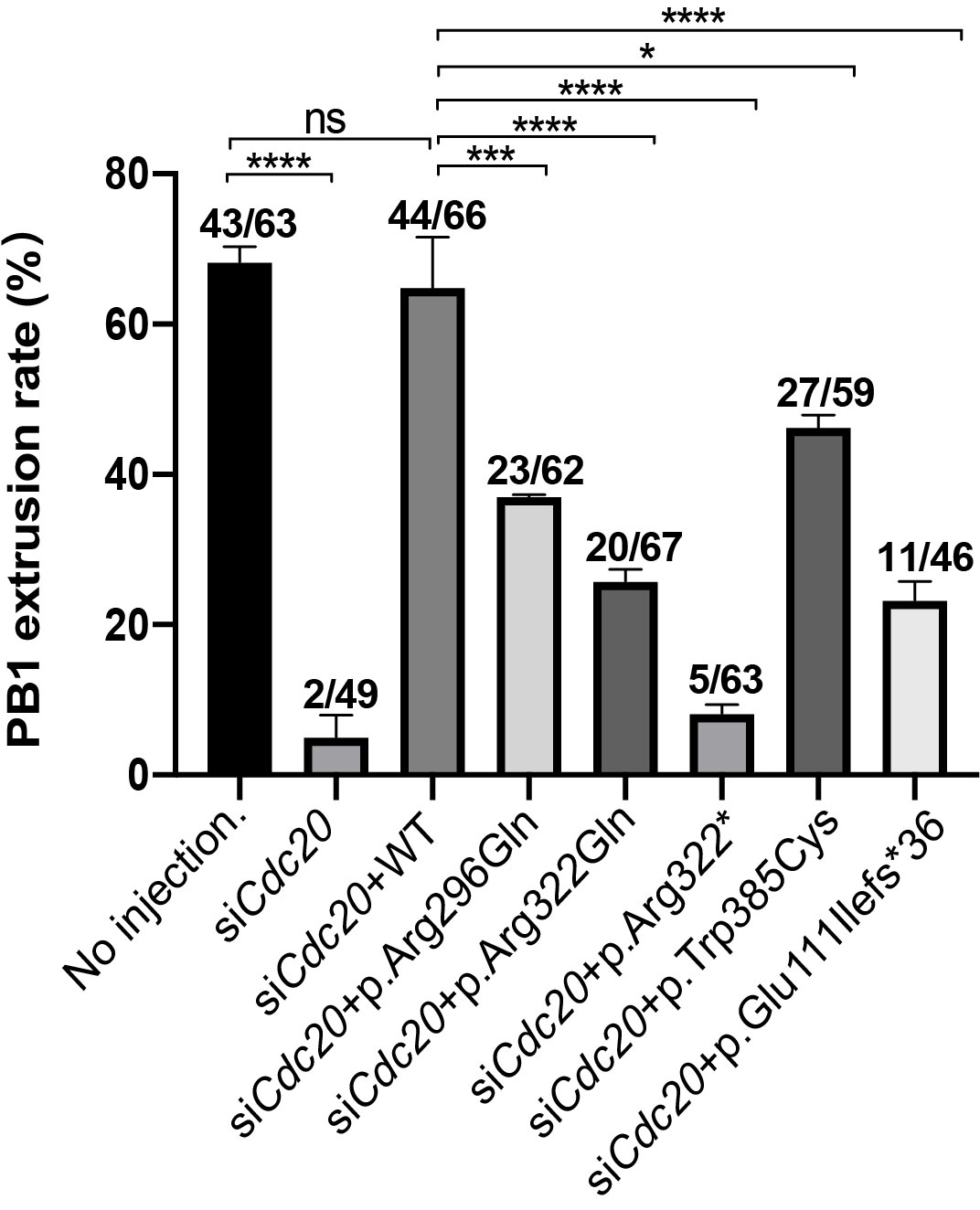

Supplement: Supplementary Figure 3 — Effects of the individual mutants on CDC20 rescue ability in Cdc20 knockdown mouse oocytes. The number of oocytes with PB1 extrusion and the total number of oocytes used are listed at the top of the column. Significance was compared between the no injection group and the siCdc20 group, the siCdc20 coupled with wild-type CDC20 cRNA group, and the siCdc20 coupled with mutant CDC20 cRNA groups. Experiments were performed followed the chi-square test. *p < 0.05, ***p < 0.001, ****p < 0.0001, ns, not significant. [file Image_3.JPEG]
