# Supplementary material for: Identification of Novel Mutations in CDC20: Expanding the Mutational Spectrum for Female Infertility
Source: Front Cell Dev Biol. 2021 Apr 9;9:647130. doi: 10.3389/fcell.2021.647130 (PMC8063106; doi:10.3389/fcell.2021.647130)
Supplement: Supplementary file 4 [file Table_1.DOC]

Supplementary Material

**Figure S1. TA cloning and sequencing of patient II-1 in family 3**

Sanger sequencing confirmation of a healthy individual and patient II-1 in family 3 indicates that the compound heterozygous mutation was located on two alleles, which confirms the recessive inheritance pattern. The two mutant bases are marked in red and framed in a red box.

**Figure S2. Effects of mutations on CDC20 protein level in the rescue experiment in mouse oocyte**

(A) The effects of the mutations on CDC20 protein level by western blotting in mouse oocytes co-injected with *cdc20* siRNA and wild-type or mutant cRNA.

(B) Quantification of wild-type and mutant CDC20 corresponding of Figure S1A. Quantification was performed by measuring the band intensity of CDC20 relative to that of vinculin. The experiment was performed with two independent biological replicates yielding similar results. The data are shown as means and SEM. *p < 0.05, ** p < 0.01.

**Figure S3. Effects of the individual mutants on *CDC20* rescue ability in *Cdc20* knockdown mouse oocytes.**

The number of oocytes with PB1 extrusion and the total number of oocytes used are listed at the top of the column. Significance was compared between the no injection group and the si*Cdc20* group, the si*Cdc20* coupled with wild-type *CDC20* cRNA group, and the si*Cdc20* coupled with mutant *CDC20* cRNA groups. Experiments were performed followed the chi-square test. * p < 0.05, *** p < 0.001, **** p < 0.0001, ns, not significant.

**Table S1. Ovarian stimulation characteristics in the three patients.**

| **Patients** | **Day** | **FSH**  **(IU/ml)** | **LH**  **(IU/ml)** | **E_2_**  **(pg/ml)** | **P**  **(ng/ml)** |
| --- | --- | --- | --- | --- | --- |
| II-1 in family 1 | MC_3_ | 7.85 | 3.53 | 53.00 | 0.43 |
|  | hCG triggering | NA | 47.68 | 1368.00 | 3.60 |
| II-1 in family 2 | MC_3_ | 3.89 | 4.89 | 76.00 | 0.20 |
|  | hCG triggering | 20.40 | 25.31 | 1066.00 | 1.80 |
| II-1 in family 3 | MC_3_ | 2.73 | 1.67 | 18.40 | 0.29 |
|  | hCG triggering | NA | 1.21 | 7235.00 | 2.02 |

MC_3_, menstrual cycle day 3; FSH, Follicle-stimulating hormone; LH, luteinizing hormone; E2, estrogen; P, progesterone; NA, not available.
